# Car Accident and Respiratory Distress: A Trauma-Based Pneumothorax Simulation Case for Medical Students

**DOI:** 10.5070/M5.52287

**Published:** 2026-07-31

**Authors:** Brandon Garten, Bradley Golden, AJ Kleinheksel

**Affiliations:** * Medical College of Georgia, Augusta, GA; ^ Medical College of Georgia, Department of Emergency Medicine, Augusta, GA; † Medical College of Georgia, Department of Medicine, Augusta, GA

## Abstract

**Audience:**

This simulation is intended for second-year medical students who are in the pre-clinical phase of their medical school curriculum. This case is delivered as part of a longitudinal simulation-based curriculum that aligns with concurrent didactic instruction in the cardiopulmonary systems course.

**Introduction:**

Pneumothorax is a potentially life-threatening condition that requires urgent attention and intervention, particularly in the setting of trauma.^1^ Traumatic pneumothorax has been reported in up to one-third of patients presenting with blunt chest wall injury.^2^ If left unrecognized or inadequately treated, this may progress to a life-threatening tension pneumothorax, resulting in severe respiratory compromise and obstructive shock with hemodynamic instability. Early familiarity with structured trauma assessment and recognition of time-sensitive cardiopulmonary deterioration represents a foundational skill for learners entering clinical training.

This case emphasizes critical actions central to emergency medicine practice, including high-flow oxygen delivery and the use of ultrasound to guide timely procedural intervention. It is also designed to introduce medical students to fundamental principles of trauma assessment and management, which are often not directly assessed in pre-clerkship curricula.^3^ Prior studies have demonstrated that early exposure to simulation-based trauma training improves learner confidence and clinical preparedness,^4^,^5^ supporting the use of this structured trauma scenario.

**Educational Objectives:**

By the end of this simulation, learners will be able to: 1) obtain a focused history in the setting of recent motor vehicle accident and respiratory difficulty, 2) identify abnormal respiratory findings consistent with pneumothorax on physical exam, 3) interpret chest x-ray and point-of-care ultrasound to identify the progression of a life-threatening tension pneumothorax,4) assess for potential internal bleeding and hypovolemic shock by integrating imaging findings, vital signs, mental status, and additional components of physical examination, 5) interpret imaging and clinical findings to guide appropriate management decisions, including need for urgent procedural intervention and escalation of care, and 6) deliver a concise oral presentation to an attending physician, including key findings and management plan.

**Educational Methods:**

This case is conducted using a high-fidelity simulation environment that includes a standardized nurse, vital sign monitoring, a mannequin, and video display for diagnostic imaging (chest x-ray and point-of-care ultrasound findings). The simulation case is structured around the ABCDE (airway, breathing, circulation, disability, exposure) approach to trauma assessment, encouraging learners to prioritize stabilization during an evolving clinical presentation.^6^ Through this scenario, learners are guided toward integrating history-taking, physical examination findings, and point-of-care diagnostics to identify and initiate management of a traumatic pneumothorax. The case also highlights core elements of acute care management including high-flow oxygen delivery, use of ultrasound, and timely coordination of procedural intervention.

This simulation was developed using the principle of instructional scaffolding, in which learners progress through a structured pathway toward key clinical objectives.^7^ For this case, scaffolding is operationalized through facilitator cues, prompts from confederate actors, and dynamic changes in patient status (vital signs and mannequin responses) based on learner interventions. This approach was chosen because it allows learners to make independent clinical decisions within a guided framework and enhances reproducibility of the case.

**Research Methods:**

This simulation was piloted with a group of medical students participating in a simulation-based elective curriculum. Learner performance was assessed using a structured critical actions checklist developed by the authors based on relevant literature in simulation-based assessment^8^ and refined by an emergency medicine physician and a simulation education specialist. The checklist was designed to capture completion of key team-based clinical actions and to facilitate targeted feedback during debriefing, consistent with established best practices in simulation-based education.^8^

The educational efficacy of the case was evaluated through structured debriefing, facilitator observation, and post-session learner feedback. Learner comments and facilitator observations were used to refine the simulation script, standardize nurse prompts, and pace the case.

**Results:**

The learner group involved in the case pilot consisted of four pre-clerkship medical students (n=4). The learner group independently completed five of seven predefined key action items, including obtaining a focused history, performing a complete physical exam, ordering point-of-care diagnostic imaging, interpreting chest radiograph findings consistent with tension pneumothorax, and delivering a concise presentation to the attending physician. Learners required prompting from confederates to escalate oxygen delivery and recognize the need for needle decompression.

Learners rated the simulation highly for clinical relevance, realism, and educational value (median of 5 on 5-point Likert scale, range 4–5). Three of four learners (75%) reported increased confidence in approaching similar scenarios and all learners (100%) reported improved understanding of FAST exam interpretation, primary trauma survey, and pneumothorax management.

**Discussion:**

This case successfully integrates clinical reasoning and team communication within a high-acuity simulation. This represents a novel addition to the medical school’s simulation curriculum because it is the first scenario in the pre-clerkship program to focus on the structured primary survey and acute management of a trauma patient. Learners benefitted from the scaffolded approach to airway assessment, application of point-of-care diagnostics, and emphasis on collaborative decision-making in a high-acuity setting.

**Topics:**

Pneumothorax, trauma, ultrasound, FAST exam, respiratory distress, primary survey, acute care, medical education, simulation.

## USER GUIDE

**Table:** 

List of Resources:	
Abstract	30
User Guide	33
Instructor Materials	37
Operator Materials	50
Debriefing and Evaluation Pearls	53
Simulation Assessment	56

**Learner Audience:**

Medical Students

**Time Required for Implementation:**

**Preparation:** 30–60 minutes for standardized actor training and tech setup.

**Time for case:** 20 minutes.

**Time for debriefing:** 20 minutes.

**Recommended Number of Learners per Instructor:** 2–4 learners per case.

**Topics:**

Pneumothorax, trauma, ultrasound, FAST exam, respiratory distress, primary survey, acute care, medical education, simulation.

**Objectives:**

By the end of this simulation, learners will be able to:

1. Obtain a focused history from a patient involved in a motor vehicle accident, identifying respiratory symptoms and mechanism of injury.
2. Perform a targeted physical examination to detect findings consistent with pneumothorax. 3. Initiate appropriate oxygen therapy based on clinical presentation and vital signs.
3. Establish IV access and initiate fluid resuscitation or blood product transfusion.
4. Order and interpret a chest radiograph and Focused Assessment with Sonography in Trauma (FAST) ultrasound exam to confirm pneumothorax and evaluate for internal bleeding.
5. Deliver a concise, structured handoff to the attending physician.
6. Explain diagnosis and treatment plan using clear, patient-centered communication.

### Linked objectives and methods

This simulation was created to provide learners with an immersive, high-acuity environment reflective of the initial emergency department evaluation of a trauma patient following a motor vehicle accident. It offers learners the opportunity to recognize clinical deterioration and stabilize a decompensating patient, identify the development of a tension pneumothorax, initiate life-saving interventions, and communicate effectively within a multidisciplinary team. Learners will prepare for the case by reviewing materials that provide a concise overview of acute management in major trauma, as well as an introduction to the use and interpretation of the eFAST ultrasound exam. The case’s educational objectives are integrated throughout the simulation. Upon entering the room, learners will obtain a focused history from a patient in respiratory distress following a motor vehicle accident (Objective 1). During the physical examination, learners will encounter unilateral decreased breath sounds with hyperresonance to percussion and visible bruising across the chest and abdomen, consistent with a seatbelt sign (Objective 2). In the setting of respiratory distress, learners are expected to initiate supplemental oxygen, which requires escalation to a non-rebreather mask for clinical improvement (Objective 3). The patient will also be hypotensive and tachycardic, prompting learners to establish intravenous access and initiate fluid resuscitation or blood product transfusion (Objective 4). Learners are expected to order diagnostic testing and interpret both chest radiograph and point-of-care ultrasound findings to identify a tension pneumothorax (Objective 5). These findings should prompt recognition of the need for emergent needle decompression to relieve intrathoracic pressure. Throughout the simulation, learners will also be expected to present the case to the attending emergency physician (Objective 6) and communicate effectively with team members, the patient, and the nurse confederate (Objective 7). Upon completion of the case, a structured debrief led by the facilitator reinforces key concepts in trauma evaluation, including important aspects of physical exam, approach to hemodynamic instability, escalation of oxygen delivery, interpretation of the FAST exam, and definitive management of pneumothorax.

This learning opportunity aligns with Kolb’s Experiential Learning Theory,^9^ which emphasizes learning through concrete experience, reflective observation, and active experimentation. This framework was selected to highlight the role of debriefing in reinforcing reflective practice and supporting the application of clinical concepts to real-world settings. This scenario also incorporates principles of instructional scaffolding, in which learners are guided through a defined set of critical actions while maintaining autonomy in decision-making, facilitated using structured prompts and patient cues. Together, these approaches have been shown to improve preparedness among pre-clerkship learners in acute care settings.^10^–^12^

### Recommended pre-reading for instructor

- PEARLS Debriefing Tool. Debrief2Learn.
https://debrief2learn.org/pearls-debriefing-tool/
  ○ Structured debriefing framework that may help facilitators review key learning points after simulation case. Includes a downloadable one-page reference and overview of the PEARLS approach.
- 5 Tips for Effective Debriefing. YouTube. Published online June 27, 2018. Accessed April 21, 2020.
https://www.youtube.com/watch?v=HEOYY2wdVRI
- Case Materials
  ○ Facilitators should review case materials prior to the simulation, including the technical setup, critical actions list, simulation events table, and core debriefing discussion points.

### Learner responsible content

- Baxman F. Major Trauma | Acute Management | (C)ABCDE. Geeky Medics. Published November 8, 2022. Accessed May 11, 2026. https://geekymedics.com/major-trauma-acute-management-cabcde/
  ○ This is a concise review of the primary trauma survey, with attention to initial interventions and red flags.
- Avila J. 5 Min Sono – E-FAST - Core Ultrasound. Core Ultrasound - free and subscription ultrasound education for medical professionals. Published October 24, 2025. Accessed April 3, 2026. https://coreultrasound.com/efast/
  ○ Brief video tutorial demonstrating FAST exam views for acute trauma assessment.

### Results and tips for successful implementation

This simulation is most optimally implemented using a high-fidelity mannequin in a dedicated simulation center, where dynamic vital signs, audiovisual imaging displays, and a standardized nurse actor are available to enhance clinical realism. The case was originally developed as part of a medical education elective focused on simulation design and instructional theory for senior medical students. Following its development, the scenario was piloted with a group of four pre-clerkship medical students (n=4) under the supervision of an interdisciplinary team that included a senior medical student, physician assistant, simulation nurse confederate actor, and course director. During the pilot, the senior year medical student who authored the case served as the facilitator, providing the patient voice and controlling changes in mannequin status throughout the scenario. The nurse confederate actor provided prompts to guide learner progression. The physician assistant observed the pilot and contributed feedback on clinical realism, while the course director assessed simulation flow and supported technical execution of the simulation equipment. This project involved the development and implementation of a simulation-based educational case for medical students. No identifiable personal data were collected, and no patient or human subject research was conducted. As such, formal ethics approval was not required.

The learner group independently completed five of seven predefined key clinical actions during the pilot simulation. Learners required prompting from confederates to appropriately escalate oxygen delivery and recognize the need for emergent needle decompression. Post-simulation feedback demonstrated high perceived clinical relevance, realism, and educational value (median 5 on a 5-point Likert scale, range 4–5). Three of four learners (75%) reported increased confidence in approaching similar scenarios, and all learners (100%) reported improved understanding of FAST exam interpretation, primary trauma survey, and pneumothorax management. Based on positive learner feedback and external faculty review by an attending emergency physician, the case was subsequently incorporated into the second-year patient-centered learning simulation curriculum.

This case was designed to be run with a group of up to four pre-clerkship learners, with each assigned a functional role: leader, gatherer, recorder, and interpreter. Roles are randomly assigned at the start of the session and are rotated across simulations to allow learners exposure to different responsibilities. The leader directs team communication, including history-taking and interaction with the patient and nurse. The gatherer brings a laptop into the simulation and uses available resources to identify relevant information (imaging, guidelines, treatment considerations). The recorder documents key elements of the encounter and later prepares a note consisting of a subjective patient History of Present Illness (HPI), objective physical exam, lab findings, and overall assessment and plan for patient management. The interpreter integrates information from all roles and freely moves between team members to support clinical reasoning and shared situational awareness. This format supports team-based clinical reasoning and helps learners practice collaboration, task delegation, and communication under pressure.

Effectiveness was assessed through structured debriefing and qualitative learner feedback following the pilot simulation. Students reported that the scenario effectively challenged their diagnostic reasoning and clinical decision-making skills. They consistently noted that the progressively worsening vital signs increased their perceived stress and prompted more urgent decision-making. Managing multiple tasks simultaneously (obtaining history while initiating interventions) was a key challenge. Additionally, learners were unsure how to appropriately escalate oxygen therapy and required additional prompting from the nurse confederate. Revisions made based on feedback included extending the case timeline, incorporating distracting symptoms to encourage a more comprehensive workup, and embedding additional nurse prompts. The patient script was also adjusted to provide opportunities for learners to demonstrate patient-centered communication during acute care encounters. This included prompts from the patient that elicit expressions of fear and concern, as well as a corresponding critical action requiring learners to explain the clinical situation and address patient concerns.

## Supplementary Information



## Figures and Tables

**Table:** 

**#1**	**FAST Exam** – Right Upper Quadrant (normal)
**#2**	**FAST exam** – Left Upper Quadrant (normal)
**#3**	**FAST exam** – Pelvis (normal)
**#4**	**FAST exam** – Subxiphoid Cardiac View (normal)
**#5**	**Right Lung** – Sliding present (normal)
**#6**	**Left Lung** – Absence of lung sliding, consistent with pneumothorax
**#7**	**Chest x-ray** – Left-sided pneumothorax with mediastinal shift

**Table:** 

**Vitals:**	Heart Rate (HR) 115	Blood Pressure (BP) 90/60
	Respiratory Rate (RR) 32	Temperature (T) 37°C
	Oxygen Saturation (O_2_Sat) 84%	

**Figure:**
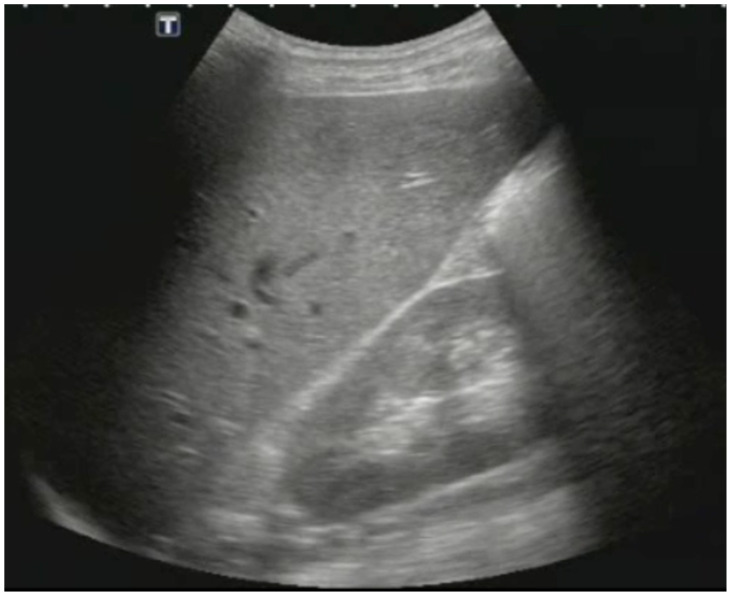


**Figure:**
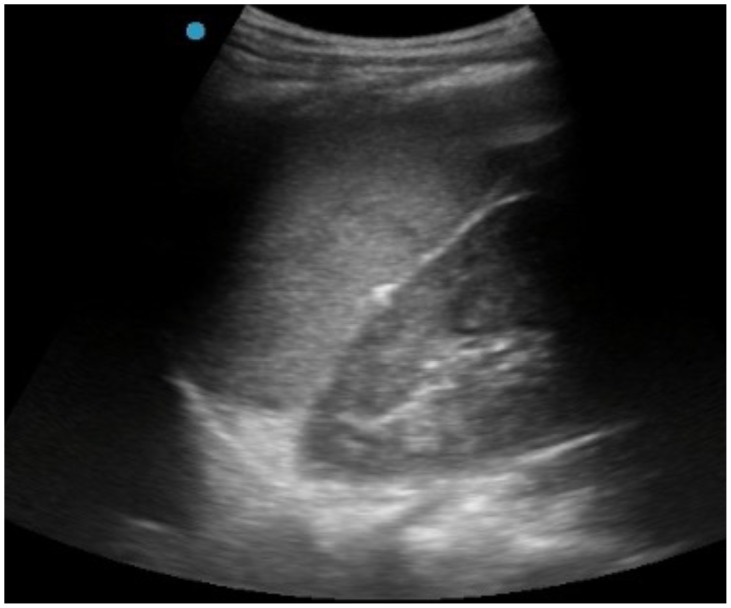


**Figure:**
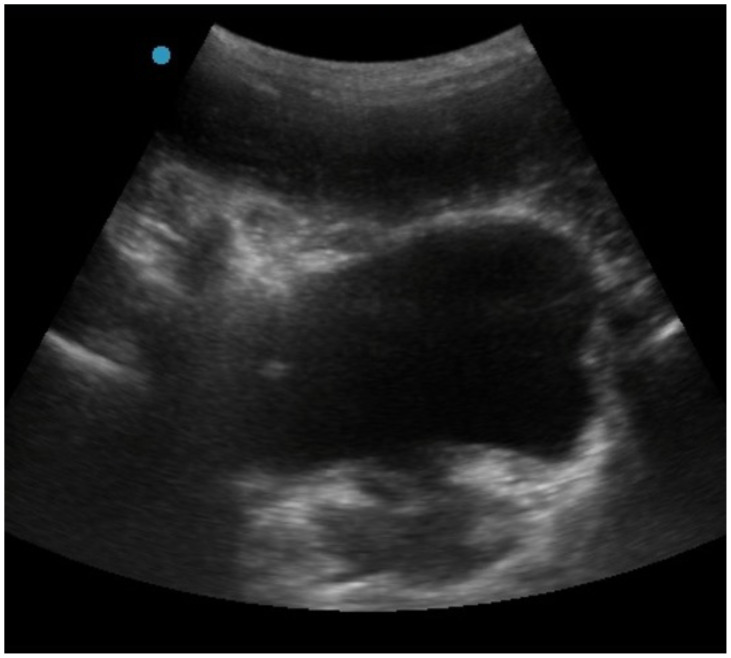


**Figure:**
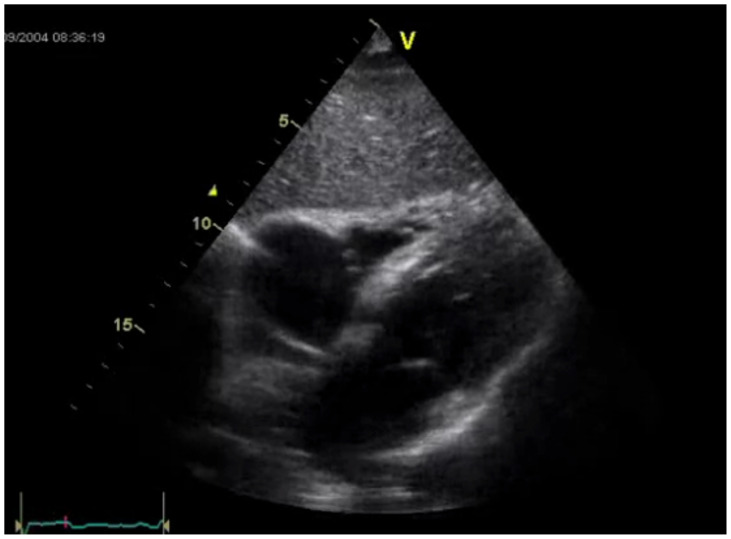


**Figure:**
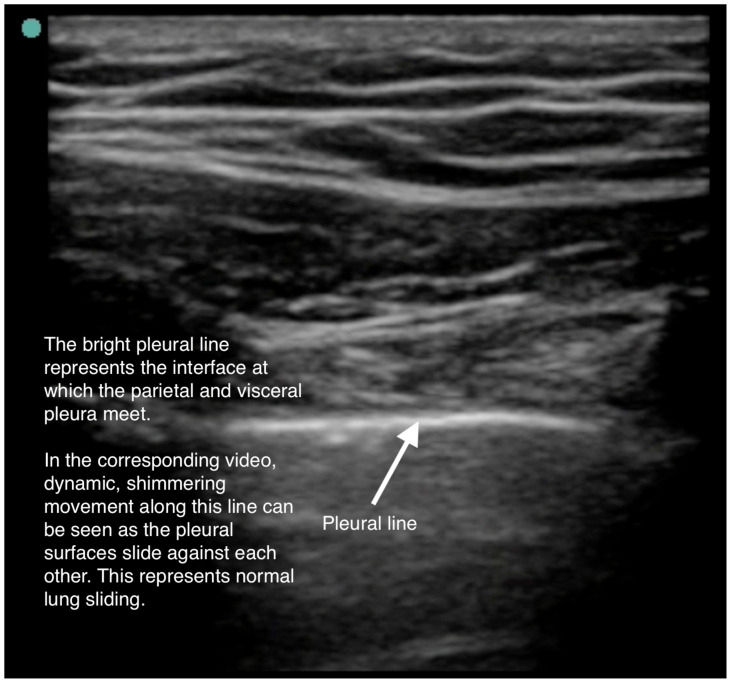


**Figure:**
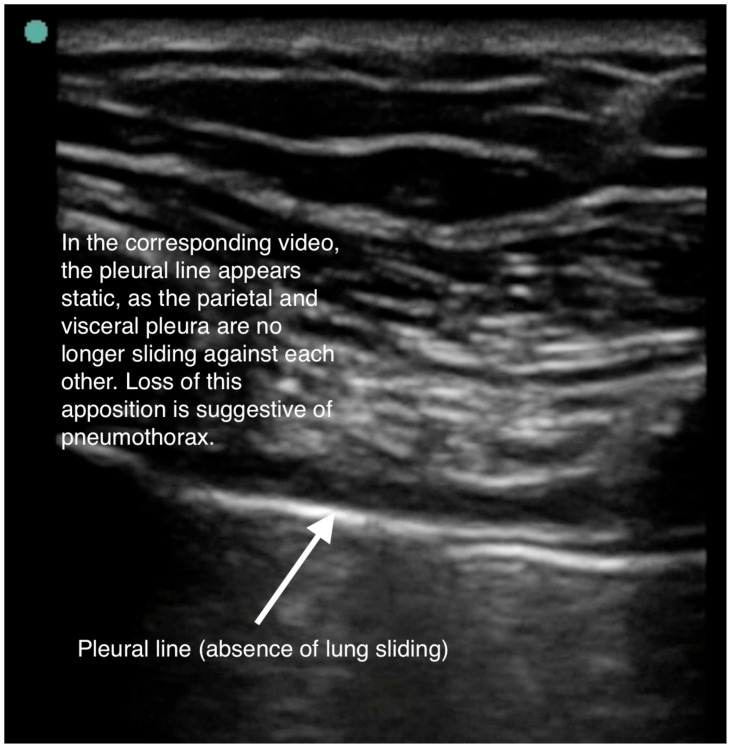


**Figure:**
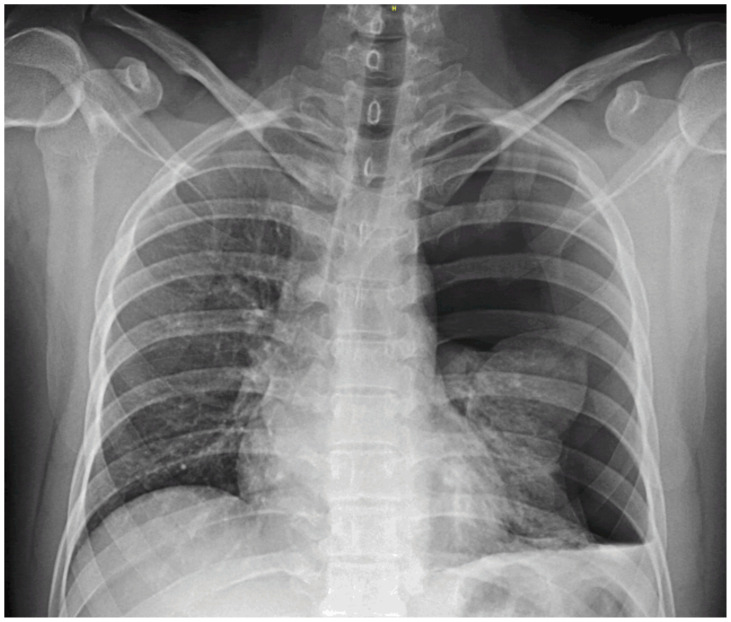


**Table:** SIMULATION EVENTS TABLE:

Minute (state)	Participant action/trigger	Patient status (simulator response) & operator prompts	Monitor display (vital signs)
0:00 (Baseline)	Learners enter, begin assessment	Patient anxious, breathing rapidly. Nurse introduces self and states patient was just brought in by emergency medical services after MVA.	T: 37C HR: 115 BP: 90/60 RR: 32 O2: 84%
1:00	Learners initiate focused history and exam	Patient reports chest/abdomen pain, worse with deep breaths. Requests help with breathing.	(same as above)
3:00	Learners start O2 with nasal cannula.	Patient’s vitals do not improve. If learners stall, nurse may prompt: “Would you like to try something more effective?”	T: 37°C HR: 115 BP: 90/60 RR: 28 O2: 86%
4:00	Learners escalate to non-rebreather on high flow.	Oxygenation mildly improves.	T: 37°C HR: 115 BP: 90/60 RR: 22 O2: 94%
5:00	Learners have not initiated IV access.	Patient remains hypotensive. Nurse may prompt: “Would you like to get IV access? We can start fluids or consider a transfusion.”	(same as above)
7:00	Learners establish IV access and give fluid bolus (or blood products).	Blood pressure modestly improves. If IV access was delayed, patient remains hypotensive until fluid bolus/blood products are initiated.	T: 37°C HR: 100 BP: 100/75 RR: 24 O2: 94%
8:00	Learners order chest radiograph.	Nurse says imaging results are delayed, encourages use of ultrasound. (Learners must view ultrasound prior to chest x-ray).	(same as above)
10:00	Learners complete and interpret FAST exam.	Nurse says that chest x-ray is now available, shows image on screen.	(same as above)
15:00	Learners interpret chest x-ray.	If learners do not progress toward consultation, Nurse may challenge learner: “What do you think is going on? Would you like to call for help?”	(same as above)
16:00	Learners initiate phone call for assistance.	Facilitator voicing as attending: “Is this urgent? What procedure do you think we should do?”	(same as above)
17:00	Learners communicate diagnosis and plan for patient to attending.	Nurse prompts learners about patient disposition: “Do you think we should admit him or observe him in the ED?” “Are there any additional labs or imaging you would like to order?”	(same as above)
18:00	Learners determine admission and additional workup.	Patient nervously asks: “What is happening to me? Why do I need to stay in the hospital?”	(same as above)
20:00 (Case Completion)	Learners communicate diagnosis, management plan, and need for admission to the patient.	End case	(same as above)

**Table:** 

**Critical Actions:** *Collects a focused history of present illness* Learners are expected to obtain key elements of the history including current symptoms, timing of symptom onset, aggravating/relieving factors, mechanism of injury, seatbelt use, relevant past medical history, and anticoagulant use. *Performs a focused trauma secondary survey (physical examination)* The secondary survey should include: Baseline cognitive assessment for alertness/mental status. Cardiac auscultation and pulmonary exam (including auscultation and percussion). Abdominal observation/palpation. Evaluation of peripheral pulses/perfusion. Assessment for cervical spine and thoracolumbar tenderness. Inspection and palpation of all extremities for signs of traumatic injury. Neurological screening, including spontaneous movement and intact sensation in all four extremities. *Initiates IV access and either fluid or blood products* *Initiates and appropriately escalates supplemental oxygen* Begins oxygen delivery via nasal cannula during initial trauma assessment. Recognizes persistent hypoxemia and escalates oxygen delivery to a higher-flow modality (non-rebreather mask). *Facilitator note: Learner may initially attempt to increase nasal cannula flow, which is acceptable. The patient will remain non-responsive to this adjustment, so the learner will need to transition to a modality that is capable of delivering >10 L/min.* *Orders and interprets point-of-care ultrasound* *Orders and interprets chest radiograph* *Recognizes pneumothorax based on clinical and imaging findings.* *Calls for assistance (ED attending or trauma team) for procedural management (needle decompression followed by definitive chest tube placement)* Learner delivers concise presentation and proposes next steps in management. *Determines appropriate disposition and additional workup* *a.* Recommends hospital admission to trauma surgery for further evaluation and close monitoring; suggests additional labs/imaging. *Communicates diagnosis and management plan with patient and team* Explains diagnosis, need for intervention, and plan for hospital admission.	0:00

**Table:** 

	Milestone	Did not achieve level 1	Level 1	Level 2	Level 3
1	**Emergency Stabilization (PC1)**	□ Did not achieve Level 1	□ Recognizes abnormal vital signs	□ Recognizes an unstable patient, requiring intervention Performs primary assessment Discerns data to formulate a diagnostic impression/plan	□ Manages and prioritizes critical actions in a critically ill patient Reassesses after implementing a stabilizing intervention
2	**Performance of focused history and physical (PC2)**	□ Did not achieve Level 1	□ Performs a reliable, comprehensive history and physical exam	□ Performs and communicates a focused history and physical exam based on chief complaint and urgent issues	□ Prioritizes essential components of history and physical exam given dynamic circumstances
3	**Diagnostic studies (PC3)**	□ Did not achieve Level 1	□ Determines the necessity of diagnostic studies	□ Orders appropriate diagnostic studies. Performs appropriate bedside diagnostic studies/procedures	□ Prioritizes essential testing Interprets results of diagnostic studies Reviews risks, benefits, contraindications, and alternatives to a diagnostic study or procedure
4	**Diagnosis (PC4)**	□ Did not achieve Level 1	□ Considers a list of potential diagnoses	□ Considers an appropriate list of potential diagnosis May or may not make correct diagnosis	□ Makes the appropriate diagnosis Considers other potential diagnoses, avoiding premature closure
5	**Pharmacotherapy (PC5)**	□ Did not achieve Level 1	□ Asks patient for drug allergies	□ Selects an medication for therapeutic intervention, consider potential adverse effects	□ Selects the most appropriate medication and understands mechanism of action, effect, and potential side effects Considers and recognizes drug-drug interactions
6	**Observation and reassessment (PC6)**	□ Did not achieve Level 1	□ Reevaluates patient at least one time during case	□ Reevaluates patient after most therapeutic interventions	□ Consistently evaluates the effectiveness of therapies at appropriate intervals
7	**Disposition (PC7)**	□ Did not achieve Level 1	□ Appropriately selects whether to admit or discharge the patient	□ Appropriately selects whether to admit or discharge Involves the expertise of some of the appropriate specialists	□ Educates the patient appropriately about their disposition Assigns patient to an appropriate level of care (ICU/Tele/Floor) Involves expertise of all appropriate specialists
9	**General Approach to Procedures (PC9)**	□ Did not achieve Level 1	□ Identifies pertinent anatomy and physiology for a procedure Uses appropriate Universal Precautions	□ Obtains informed consent Knows indications, contraindications, anatomic landmarks, equipment, anesthetic and procedural technique, and potential complications for common ED procedures	□ Determines a back-up strategy if initial attempts are unsuccessful Correctly interprets results of diagnostic procedure
20	**Professional Values (PROF1)**	□ Did not achieve Level 1	□ Demonstrates caring, honest behavior	□ Exhibits compassion, respect, sensitivity and responsiveness	□ Develops alternative care plans when patients’ personal beliefs and decisions preclude standard care
22	**Patient centered communication (ICS1)**	□ Did not achieve level 1	□ Establishes rapport and demonstrates empathy to patient (and family) Listens effectively	□ Elicits patient’s reason for seeking health care	□ Manages patient expectations in a manner that minimizes potential for stress, conflict, and misunderstanding. Effectively communicates with vulnerable populations, (at risk patients and families)
23	**Team management (ICS2)**	□ Did not achieve level 1	□ Recognizes other members of the patient care team during case (nurse, techs)	□ Communicates pertinent information to other healthcare colleagues	□ Communicates a clear, succinct, and appropriate handoff with specialists and other colleagues Communicates effectively with ancillary staff

## INSTRUCTOR MATERIALS

**Case Title:** Car Accident and Respiratory Distress

**Case Description & Diagnosis (short synopsis):** 25-year-old male presents with shortness of breath, nonspecific abdominal/chest pain after a motor vehicle collision (MVC). He is found to have a left-sided tension pneumothorax requiring urgent recognition and intervention.

**Equipment or Props Needed:**

High-fidelity mannequin, vital sign monitor, display screen for ultrasound images and chest X-ray, non-rebreather facemask, oxygen source with tubing, IV start kit (with angiocath and tubing), intravenous (IV) fluids (eg, 0.9% Normal Saline), packed red blood cells, IV poles, and moulage (seatbelt sign or bruising across chest/abdomen of mannequin).

*If a high-fidelity mannequin is not available, this simulation may be adapted using a low-fidelity mannequin or standardized patient. In this setting, vital signs, physical examination findings, and changes in clinical status can be communicated verbally by the facilitator or nurse confederate. Imaging studies (eg, ultrasound clips and chest radiographs) can be displayed on a shared screen or provided as printed materials to maintain key learning objectives.

**Embedded participants needed:**

1. **Nurse:** An actor who presents diagnostic tests/images, performs closed-loop communication, and prompts learners to progress along simulation. The nurse confederate should be provided with the case materials in advance, including the full scenario, simulation events table, and scripted prompts, to ensure familiarity with the case and allow for consistent guidance of learner performance.
2. **Facilitator (patient voice actor):** Serves as the patient voice and controls changes in mannequin status and overall case progression from an external control room. The case facilitator should ideally be either a physician or senior medical student familiar with the clinical content of the case. The facilitator responds to learner questions using the information provided in the case materials (history and review of systems) and structured prompts within the simulation events table. Responses should remain consistent with the patient’s evolving clinical condition.

**Stimulus Inventory:**

**Background and brief information:** Patient was brought to the local emergency department by ambulance after being rear-ended in a motor vehicle accident.

**Initial presentation:** Patient is alert but visibly distressed, diaphoretic, with increased work of breathing.

**How the scenario unfolds:** The case begins with a young adult presenting with chest and upper abdominal pain following a recent motor vehicle collision. When asked, the patient reports being the restrained driver in a rear-end collision occurring 30 minutes prior to arrival. He describes sudden onset left-sided chest pain and shortness of breath that worsens with deep inspiration. Learners are expected to obtain this focused history while initiating a structured primary trauma survey. The patient also presents with tachycardia, borderline hypotension, tachypnea, and hypoxemia, prompting learners to initiate supplemental oxygen therapy. If only minimal interventions are attempted (eg, nasal cannula), the patient’s oxygen saturation will remain low, prompting the need for escalation to high-flow oxygen delivery via a non-rebreather mask.

Learners are expected to establish intravenous access (IV), as well as initiate fluid resuscitation and/or blood product transfusion given the patient’s hemodynamic instability and visible seatbelt sign, which is suggestive for potential intra-abdominal bleeding. If learners do not initiate IV access during the primary survey, the nurse confederate may prompt the team by asking whether vascular access should be obtained. Once IV access is established, learners may administer an initial fluid bolus (eg, 500 mL normal saline) or transfusion of packed red blood cells. This intervention will modestly improve the patient’s blood pressure. To progress the case, learners should suggest bedside imaging, including a Focused Assessment with Sonography in Trauma (FAST) examination and obtain a chest X-ray. These images will be available to view on monitors in the simulation room.

The lung portion of the FAST exam will display absence of lung sliding on the left, while the additional right upper quadrant, left upper quadrant, and subxiphoid views will not show evidence of intra-abdominal or pericardial bleeding.

The FAST exam is intentionally prioritized as the first bedside diagnostic test in the setting of trauma. If learners attempt to order labs or a chest X-ray prior to the FAST exam, the nurse will let them know these tests are pending and prompt them by asking, “Is there anything else you want to do at the bedside?” This redirection will encourage learners to consider ultrasound early.

Once the pneumothorax is identified, learners are expected to consult their attending emergency physician to articulate the identification of a tension pneumothorax on chest radiograph and the need for procedural intervention. Procedural management may include urgent needle decompression to temporize suspected tension physiology, followed by definitive chest tube placement to facilitate lung re-expansion and allow evacuation of a potential intrathoracic bleed. After the consultation, both the nurse and the patient will prompt the learner to clearly communicate the diagnosis, explain the treatment plan to a fearful patient, and begin discussing next steps in care. The case concludes after learners outline an appropriate disposition plan, including additional imaging and admission to the trauma surgery service.

**Critical actions:**

1. Collects a focused history of present illness.
2. Performs a focused trauma secondary survey (physical examination).
3. Initiates IV access and either fluid or blood products.
4. Initiates and appropriately escalates supplemental oxygen.
5. Orders and interpret point-of-care ultrasound.
6. Orders and interpret chest radiograph.
7. Recognizes pneumothorax based on clinical and imaging findings.
8. Consults an appropriate provider (ED attending or trauma team) for procedural management (needle decompression followed by definitive chest tube placement).
9. Determines appropriate disposition and additional workup.
10. Communicate diagnosis and management plant with patient and team.

**Case Title:** Car Accident and Respiratory Distress

**Chief Complaint:** “It is hard to breath; my chest and stomach hurt too.”

**General Appearance:** Patient is alert but visibly anxious and diaphoretic, with increased work of breathing

**Primary Survey:**

- **Airway:** Intact
- **Breathing:** Labored
- **Circulation:** Mild hypotension, tachycardia

**History:**

- **History of present illness:** Sudden onset chest and abdominal pain shortly after MVC, worsened by deep breathing, with radiation to the back.
- **Review of systems:**
  ○ Neurologic: Denies loss of consciousness, headache, focal weakness, or vision changes.
  ○ Cardiovascular: Reports lightheadedness and racing heartbeat.
  ○ Respiratory: Endorses shortness of breath and sharp, left-sided chest pain with inspiration. Denies hemoptysis.
  ○ Gastrointestinal: Reports diffuse abdominal tenderness, denies nausea or vomiting.
- **Past medical history:** 25-year-old male with no significant medical history
- **Past surgical history:** Appendectomy (4 years ago)
- **Patient’s medications:** None
- **Allergies:** No Known Drug Allergies
- **Social history:** Lives in apartment with friend, in grad school, drinks 2–3 alcoholic beverages per week, does not smoke/vape, no illicit drug use.
- **Family history:** Father has HTN, mother has Type II Diabetes.

**Secondary Survey/Physical Examination:**

- **General appearance:** Anxious, diaphoretic young male in moderate respiratory distress.
- **HEENT:**
  ○ **Head:** No scalp lacerations or obvious signs of external head trauma.
  ○ **Eyes:** Pupils equal and reactive.
  ○ **Ears:** within normal limits
  ○ **Nose:** within normal limits
  ○ **Throat:** within normal limits
- **Neck:** Trachea midline. No midline cervical spine tenderness. No jugular venous distension.
- **Heart:** Tachycardic with regular rhythm. Peripheral pulses palpable.
- **Lungs:** Tachypneic with increased work of breathing. *Decreased breath sounds on left thorax* with *hyperresonance to percussion*. Normal breath sounds on right. Tenderness to palpation over left anterior chest wall without crepitus.
  ○ These findings may be provided by the nurse if the learner specifically performs lung auscultation and and/or attempts percussion.
- **Abdominal/GI:** Tenderness to deep palpation of epigastric region. No distention or rebound tenderness.
- **Genitourinary:** No visible blood at urethral meatus.
- **Rectal:** Normal tone. Negative for blood.
- **Extremities:** No gross deformities, tenderness, swelling, or external signs of trauma in all four extremities. Patient moves all extremities spontaneously.
- **Back:** No thoracic or lumbar midline tenderness or step-offs.
- **Neuro:** Alert and oriented x3. No focal motor deficits. Patient has intact sensation in all four extremities.
- **Skin:** Bruising over upper chest and abdomen (seatbelt sign).
  ○ *If a high-fidelity mannequin is not available, the nurse confederate or facilitator should verbally provide notable exam findings above.

*Still images are shown below. Corresponding ultrasound video clips are included in the stimulus inventory where available.*

*FAST Exam – Right Upper Quadrant (normal)*

Source: JETem. Available at: https://jetem.org/morrisons-pouch-us-2/. Published November 5, 2016. Accessed August 25, 2025. Original License CC-BY 4.0.

*FAST exam – Left Upper Quadrant (normal)*

Nguyen CM, Hartmann K, Goodmurphy C, Flamm A. E-FAST Ultrasound Training Curriculum for Prehospital Emergency Medical Service (EMS) Clinicians. *J Educ Teach Emerg Med*. 2024;9(1):C41–C97. Published January 31, 2024. Accessed August 25, 2025. Original License CC-BY 4.0. doi:10.21980/J8S060

*FAST exam – Pelvis (normal)*

Nguyen CM, Hartmann K, Goodmurphy C, Flamm A. E-FAST Ultrasound Training Curriculum for Prehospital Emergency Medical Service (EMS) Clinicians. *J Educ Teach Emerg Med*. 2024;9(1):C41–C97. Published January 31, 2024. Accessed August 25, 2025. Original License CC-BY 4.0. doi:10.21980/J8S060

*FAST exam – Subxiphoid Cardiac View (normal)*

CardioNetworks, Public domain, via Wikimedia Commons. Available at: https://commons.wikimedia.org/wiki/File:Subcostal_normal_(CardioNetworks_ECHOpedia).w ebm Published January 31, 2024. Accessed August 25, 2025. Original License CC-BY 4.0.

*Right Lung – Sliding present (normal)*

Nguyen CM, Hartmann K, Goodmurphy C, Flamm A. E-FAST Ultrasound Training Curriculum for Prehospital Emergency Medical Service (EMS) Clinicians. *J Educ Teach Emerg Med*. 2024;9(1):C41–C97. Published January 31, 2024. Accessed August 25, 2025. Original License CC-BY 4.0. doi:10.21980/J8S060

*Left Lung – Absence of lung sliding, consistent with pneumothorax*

Nguyen CM, Hartmann K, Goodmurphy C, Flamm A. E-FAST Ultrasound Training Curriculum for Prehospital Emergency Medical Service (EMS) Clinicians. *J Educ Teach Emerg Med*. 2024;9(1):C41–C97. Published January 31, 2024. Accessed August 25, 2025. Original License CC-BY 4.0. doi:10.21980/J8S060

*Chest x-ray – Left-sided pneumothorax with mediastinal shift*

Easvur K. Chest x-ray with left-sided pneumothorax and mediastinal shift. In: Wikimedia Commons. https://commons.wikimedia.org/wiki/File:Pneumothorax_gif_1.gif. Published April 11, 2016. Accessed April 20, 2026. riginal License Public Domain, Creative Commons Attribution-Share Alike 3.0 Unported license.

## OPERATOR MATERIALS

**Diagnosis:** Left-sided tension pneumothorax

**Disposition:** After decompressive procedure in ED, the patient should be admitted to the trauma surgery service given the mechanism of injury, potential for internal bleeding, and need for tube thoracostomy. In this high-acuity setting, the patient will be expedited to CT, and labs will be drawn immediately. A CT of the chest and abdomen is important to monitor for pneumothorax resolution and assess for additional intra-abdominal injuries. Helpful laboratory studies may include complete blood count, comprehensive metabolic panel, lactate, type and screen, and coagulation panel. EKG should also be obtained, especially if there is concern for cardiac contusion.

## DEBRIEFING AND EVALUATION PEARLS

### Car Accident and Respiratory Distress: A Trauma-Based Pneumothorax Simulation Case for Medical Students

**Core Debriefing Discussion Points:**

This case is designed to reinforce the skills required for evaluation of a patient following a motor vehicle collision. The debriefing session should be led by the case facilitator, who is typically a physician or senior medical student familiar with the case content. Learners should reflect on the following clinical takeaways:

- Initiate a structured primary survey in all trauma patients and pay special attention to those demonstrating borderline physiologic abnormalities. Even subtle changes in vital signs may represent early clinical decompensation. In this case, the patient arrived with clear evidence of hemodynamic instability requiring prompt management. Prioritization of airway, breathing, and circulation is essential, and early recognition of deterioration remains critical in trauma care.^13^
  ○ The primary survey consists of a rapid assessment to identify and treat life-threatening injuries using the ABCDE approach (airway, breathing, circulation, disability, exposures).^14^
  ○ Practical methods for assessment include asking patient to evaluate airway patency. Breathing is assessed with inspection of chest rise, palpation, auscultation. Circulation should focus on hemorrhage control and securing IV access with fluid or blood product resuscitation in the setting of hemodynamic instability or suspected bleeding. Disability can be assessed through the Glasgow Coma Scale (GCS) to evaluate gross neurological status. Lastly, exposures are assessed by fully undressing patient to identify occult injuries.
- When performing a target physical examination, it is important to systematically assess for injuries associated with trauma. This includes baseline cognitive assessment for alertness and mental status, cardiac and pulmonary examination (including auscultation and percussion), abdominal inspection and palpation, evaluation of peripheral perfusion, assessment for cervical spine and thoracolumbar tenderness, inspection and palpation of all extremities for signs of traumatic injury, and focused neurological screening of motor and sensory function for all four extremities.^15^
- Utilize point-of-care ultrasound (FAST exam) as a first-line bedside diagnostic test in the evaluation of blunt trauma, particularly in the setting of hemodynamic instability. Key views include the right upper quadrant, left upper quadrant, subxiphoid, and bilateral anterior lung windows. The FAST exam can rapidly identify intra-abdominal or pericardial bleeding, as well as absence of lung sliding, to guide early intervention.^16^
- Recognize when to appropriately escalate oxygen delivery in patients who do not improve with initial interventions.
  ○ Aim for maintaining oxygen saturation at 94% or higher for non-intubated patients.^17^
  ○ In alert patients, noninvasive oxygen delivery options include nasal cannula, face mask, high-flow nasal oxygen, and BiPAP/CPAP.^18^
  ○ Escalation should begin with low-flow devices and increase stepwise based on patient response.
  ○ Nasal cannula is generally limited to 6 L/min, whereas non-rebreather can deliver up to 15 L/min with an FiO2 of 60–90% depending on mask seal.
  ○ High-flow nasal oxygen can deliver flows up to ~50 L/min and may be used in patients with persistent hypoxemia who are able to protect their airway.
  ○ BiPAP/CPAP can deliver higher flow rates but should be avoided in patients at risk for aspiration or with altered mental status.
- Distinguish between needle decompression and tube thoracostomy in the management of pneumothorax.
  ○ Learners should understand that needle decompression is a temporizing intervention indicated for tension pneumothorax with hemodynamic instability, providing rapid pressure relief of air compressing the heart and lungs. Risks of needle decompression include potential injury to nearby structures including the intercostal neurovascular bundle, as well as direct damage to lung parenchyma.^19^
  ○ Tube thoracostomy is definitive management for pneumothorax, allowing continuous evacuation of air and re-expansion of the lung. If needle decompression is performed first, chest tube placement should follow for ongoing drainage and prevention of recurrence. Chest tube placement carries risks such as infection, bleeding, and injury to surrounding structures.^20^
- Practice concise, closed-loop communication with team members and consultants to ensure effective coordinated care. This includes delivery of clear handoffs, acknowledging instructions, and confirming shared understanding among members during high-acuity scenarios.
- Demonstrate compassionate, patient-centered communication when interacting with all patients, including those experiencing distress during acute trauma evaluation. In the setting of acute trauma evaluation, learners should acknowledge fear, provide clear and simple explanations, offer reassurance, and maintain effective communication while continuing time-sensitive clinical assessment.

**Other debriefing points:**

- If learners delayed oxygen escalation, ask: “What clues in the vitals or patient presentation indicated the need to act sooner?”
- If learners suggested other imaging before using ultrasound, ask: “What are the benefits of doing bedside imaging first? What might have been missed if you waited for formal studies?”
- If learners did not communicate the diagnosis to the patient, ask: “How might hearing silence from the team impact a patient in distress? What are simple ways to reassure a concerned trauma patient?”
- Encourage learners to reflect on how the patient’s subjective complaints (chest and abdominal pain) influenced their differential diagnosis and diagnostic workup. Learners should consider how these symptoms may represent overlapping pathologies (cardiac contusion, rib fractures, splenic laceration, etc.)

**Wrap Up:**

Facilitators may conclude with a brief discussion of additional concepts covering:

- Pneumothorax recognition and management - https://www.emdocs.net/em3am-pneumothorax/#:~:text=Management
- Escalating oxygen delivery methods in the emergency department - https://opencriticalcare.org/encyclopedia/overview-of-oxygen-delivery-devices/#:~:text=LOW%20FLOW%20DELIVERY%20DEVICES
- Key elements of trauma team communication and handoff - https://scholarsarchive.byu.edu/studentpub/362/#:~:text=members%20should%20use%20closed,and%20direction%20from%20the%20beginning

Optional scoring rubric for pneumothorax case (attached)

## SIMULATION ASSESSMENT

*Car Accident and Respiratory Distress*

Learner: _________________________________________

***Assessment Timeline***

This timeline is to help observers assess their learners. It allows observer to make notes on when learners performed various tasks, which can help guide debriefing discussion.

**Critical Actions:**

□ Collects a focused history of present illness.
□ Performs a focused trauma secondary survey (physical examination).
□ Initiates IV access and either fluid or blood products.
□ Initiates and appropriately escalates supplemental oxygen.
□ Orders and interpret point-of-care ultrasound.
□ Orders and interpret chest radiograph.
□ Recognizes pneumothorax based on clinical and imaging findings.
□ Consults an appropriate provider (ED attending or trauma team) for procedural management (needle decompression followed by definitive chest tube placement).
□ Determines appropriate disposition and additional workup.
□ Communicate diagnosis and management plant with patient and team.

**Summative and formative comments:**

**Milestones assessment:**
